# Health care professionals’ experiences with goal setting during initial rehabilitation after newly acquired spinal cord injury/ disorder – a qualitative focus group study

**DOI:** 10.3389/fresc.2022.982321

**Published:** 2022-08-18

**Authors:** Patricia Lampart, Livia Schäppi, Wolf Axel Langewitz, Sara Rubinelli, Diana Sigrist-Nix, Anke Scheel-Sailer

**Affiliations:** ^1^ Swiss Paraplegic Center, Nottwil, Switzerland; ^2^ Department of Health Sciences and Medicine, University of Lucerne, Lucerne, Switzerland; ^3^ Psychosomatic Medicine, University Hospital Basel, Basel, Switzerland; ^4^ Swiss Paraplegic Research, Nottwil, Switzerland

**Keywords:** goal setting (GS), organizational objectives, rehabilitation, spinal cord injuries (MeSH), health care professionals (HCP), patient care team, health personnel (MeSH), focus group (MeSH d017144)

## Abstract

**Introduction:**

Goal setting (GS) is an important aspect of initial spinal cord injury/ disorder (SCI/D) rehabilitation. However, because expected outcomes are individual and often difficult to determine, GS is not straightforward. The aim of this study was to explore the health care professionals' (HCP's) experiences with and perspectives on the goal-setting process (GSP) during initial SCI/D rehabilitation.

**Method:**

Five semi-structured focus groups (FG) (22 purposively sampled HCP, mostly in leadership positions, six different professions). The FG were transcribed verbatim. We analyzed the transcripts for qualitative content analysis following Braun and Clarke (2013).

**Results:**

HCP described GS-influencing aspects at the macro, meso and micro levels. At the macro level, participants spoke about restrictions imposed by health insurers or difficulties in planning the post-inpatient setting. Regarding the meso level, HCP spoke of institutional structures and culture that facilitated the GSP. At the micro level, knowledge of the diagnosis, expected outcomes, and individual patient characteristics were mentioned as important to the rehabilitation process. It was important for HCP to be patient and empathetic, to endure negative emotions, to accept that patients need time to adjust to their new situation, and to ensure that they do not lose hope. Open communication and interprofessional collaboration helped overcome barriers in the GSP.

**Discussion:**

This paper shows the complex relationship between external (e.g., health insurers), emotional, and communication aspects. It calls for a comprehensive approach to optimizing the GSP, so that patients' experiences can be fully considered as a basis to identify the most appropriate care pathway.

## Introduction

Spinal cord injury/ disorder (SCI/D) causes varying degrees of impairment depending on the level of the lesion (1). Comprehensive rehabilitation based on the International Classification of Functioning, disability and health (ICF) is recommended to regain an optimal level of functioning in interaction with the patient's environment (2).

Goal setting (GS) is part of the underlying construct of rehabilitation; the Rehab-Cycle© (3, 4). The Rehab-Cycle© is an ICF-based management tool. It consists of four procedural elements: assessment, assignment, intervention, and evaluation. For each of the rehabilitation cycles, goals are repeatedly set that determine assignments and appropriate interventions. Thus, GS is an essential component of the structure of a patient's rehabilitation (3). It describes the desired outcome of specific interventions and breaks down the overall goal into manageable steps (5). Therefore, in addition to motivating the team and patient, GS can coordinate and structure rehabilitation (6, 7). GS begins during acute care when the primary goal is to stabilize the patient's health (8, 9).

Ideally, rehabilitation is embedded in the work of an interprofessional team (IPT). In an IPT different health care professionals (HCP) work together to achieve common goals (10). The collaboration in the IPT guarantees different perspectives in the goal-setting process (GSP) and therefore, allows a comprehensive definition of a main goal, considering the short-term goals (6). Interprofessional collaboration improves both the patient experiences and the benefits of the rehabilitation process (11). GS also improves the patient's physical, social, and psychological adjustment to SCI/D (10). For better goal achievement it is important that patients are fully integrated into the GSP (12, 13). When HCP and patients approach the GSP as partners it helps patients adopt a positive attitude toward the GSP and achieve the best possible outcome (10, 14, 15).

GS is a multifaceted and quite complex process that is challenging for both patients and HCP (16). Previous literature describes several frameworks that provide a practical guide to the GSP. One established framework describes the five SMART (S = specific, M = measurable, A = attainable, R = realistic, T = time-bound) criteria developed by Doran (1981) (17). Ryan R. Bailey (2019) describes the importance of setting appropriate goals by considering the goal characteristics and level of difficulty. For example, in addition to a performance goal, where a person may fail, a learning goal should always be set that focuses on improving skills. In addition, the goal should be challenging but not too difficult to achieve to increase patient self-efficacy (18). To negotiate goals that are meaningful to the individual, patients need to explicitly state their problems and treatment goals (19), e.g. using the Canadian Occupational Performance Measure scale (20). However, the GSP is complex, especially in chronic conditions like SCI/D that impact many aspects of functioning and have uncertain outcomes (9, 21). To date, there is no agreement on the best approach for applying GS in daily practice in rehabilitation clinics (16). In addition, there is still little information about HCP's opinion on this topic (22).

Therefore, the aim of this study was to explore the HCP's experiences with and perspectives on the GSP during initial rehabilitation after newly acquired SCI/D. Ultimately, the findings may reveal aspects for improvement in clinical practice.

## Method

### Study design and setting

We conducted a qualitative study based on exploratory focus groups (FG) in a specialized acute care and rehabilitation center for patients with SCI/D.

In the center, rehabilitation has been based on the ICF since 2006 and described in an institutional rehabilitation concept. In this rehabilitation concept, the Rehab-Cycle© was explicitly mentioned as the underlying concept. After years of interprofessional preparatory work, the Nottwil Standard was introduced in 2019 as a clinical assessment schedule of functioning in patients after newly acquired SCI/D (23). Weekly interprofessional team meetings have been based on the ICF structure since 2006. In 2016, these team meetings were restructured and explicitly focused on GS, combined with a GS visit. Therefore, in addition to an unstructured and individually conducted GS with each patient, GS took place in two-weekly GS rounds on the ward. Initially, the HCP of the IPT (physicians, nurses, physiotherapists, occupational therapists, psychologists, and social workers) exchanged information without patients. Later, the IPT visited the patients at the GS visit. At these GS visits, the patients' status/progress was assessed and then new goals were set. Patient participation in GS was a key aim of these meetings.

To improve communication skills (both interprofessionally and between professional groups and patients) regular communication skills trainings were implemented at the center in 2014 (24). The trainings were based on the Basel communication concept (25) and were in line with the national communication guideline (26).

### Sampling and recruitment of participants

We purposively sampled the FG. Participants represented the interprofessional setting in the different wards. All participants were (1) over 18 years of age, (2) HCP, and (3) had been working in the clinic for at least six months, with most holding a leadership position. We informed the IPT of each rehabilitation ward (consisting of senior physicians, nurses, physiotherapists, occupational therapists, psychologists, and social workers) by e-mail about the study and its underlying aim. We asked them if they would like to participate with their team. All five rehabilitation wards agreed to participate. Lack of time was the reason for non-participation by single IPT members. The study followed the good clinical practice guidelines as well as the COnsolidated criteria for REporting Qualitative research (27).

### Data collection

A researcher trained in qualitative methods (PL) moderated the FG while the assistant (LS) took notes in November 2020. The FG were held in a meeting room in the clinic and were scheduled during regular ward meeting times to increase participation rate. Participants, FG leader and FG assistant were present at all meetings. No repeat FG were conducted.

We used a semi-structured FG guide (see Supplementary Table 1) focusing on previously identified aspects of the GSP (28): (1) general aspects of GS, (2) patient-specific factors, (3) HCP-specific factors, (4) organizational aspects, and (5) recommendations for improving the GSP. The FG guide was developed in collaboration with a physician experienced in rehabilitation research (AS) and experts in communication (AS, SR, WL). It was pre-tested and improved with the help of colleagues (scientific assistant, physiotherapist, psychologist). FG were held in German, audio-recorded, and transcribed verbatim by LS using a transcription and coding guide. In case of uncertainty the wording was double-checked by PL. Transcripts were not returned to participants nor did participants provide feedback on the results. FG five was used for saturation proof and no new aspects emerged.

### Data analysis

We performed coding with the MAXQDA program (Version MAXQDA Analytics Pro 2020, release version 20.3.0). Data analysis was conducted independently by two researchers (LS, PL) based on Braun and Clarke's (2013) qualitative content analysis (29). In a first step, we (LS, PL) inductively categorized the quotes and identified aspects relevant to the aim of this study. The quotes were then grouped into codes. In an iterative process, the codes were further summarized and regrouped. The codes were then discussed with the experts (AS, WL, SR, DS) to include their perspectives, which led to the definition of themes. The quotes relevant to this paper were translated into English. Descriptive analysis included participants' sex, profession, and years in the clinic.

## Results

We conducted five FG lasting approximately one hour with a total of 22 of the 30 invited participants. Instead of a senior physician, who could not attend for organizational reasons, two residents participated; none of the participants dropped out. Each FG consisted of at least four participants representing at least three different professional groups (see Supplementary Table 2). The majority of participants (82%) were female and had more than five years of professional experience in the clinic (73%). Most participants were physicians (27%) followed by psychologists (23%), physiotherapists (18%), occupational therapists (9%), and a social worker (5%) (see Supplementary Table 3).

Participants' perspectives were categorized under: (1) macro level, (2) meso level, and (3) micro level. The micro-level categories could be grouped into the three subcategories of knowledge, emotions, and communication.

### Category 1: The macro level

HCPs mentioned the influence of health insurance companies which have for example, a decisive influence on the time a patient is allowed to spend in the rehabilitation clinic. When health insurance representatives insisted on a short(er) rehabilitation period this was often perceived as having a negative impact on GS (Q1, Q2).

Several HCP stressed the importance of preparing the patient for the time of discharge; they referred to the importance of knowing what conditions the patient will live in after discharge. This included the housing situation, proximity to work and outpatient care, the willingness of family members to support the patient, etc. When patients had to be transferred to a chronic care facility with limited capacity for people with SCI/D organizing discharge was particularly difficult (Q3). In general, a scarcity of flexible solutions for accommodating a patient had an impact on the goal of reintegrating the patient into society (Q4).

### Category 2: The meso level

Participants discussed several influencing factors regarding structural elements of the rehabilitation clinic.

HCP emphasized the importance of regular interprofessional GS visits with patients and agreed that this element was very beneficial for the GSP (Q5). The GS visits provided an opportunity to focus the rehabilitation process on patients and their active participation (Q6, Q7). HCP hypothesized that the introduction of a routine GSP in 2016 (see Study design and setting) was a major reason why they had encountered fewer dissatisfied patients in recent years (Q8).

In addition, participants mentioned the benefit of other institutionalized structures that made it easier to incorporate family members' perspectives early in the rehabilitation process. This was particularly helpful for patients with challenging backgrounds and helped to better understand and interact with patients (Q9) (see Table 1).

**Table 1:** The macro and meso level.

The macro level	
Q1	« […] in these ten years that I have been here (in the clinic), it has also become worse with the health insurance companies cutting time […] Which then, like, also has a very big influence on the goals. In other words, you have to try to do faster what you were allowed nine months for before, now you have to do it in eight or seven, depending on the situation. I find that very difficult, yes, that you can still do that well. » (Social Worker 2, FG04)
Q2	« Because that also depends on the cost approval, how long a patient stays. Because the estimated time at the beginning is that a Tetra stays here, let's say nine months, a Para about six months. But depending on the insurance company and the course of the rehabilitation, this (the length of stay) can be greatly reduced. That has a big influence on the goals. » (Occupational Therapist 3, FG04)
Q3	« […] if someone has to go to a care home […] In the past, you could reserve a place in a care home […] You can't do that anymore. If it's free (the room in the care home), you have to take it, whether the assistive devices are ready or not. » (Social Worker 2, FG04)
Q4	« (comments on the lack of assisted living facilities) Because we all suffer from the fact that we cannot actually prepare the goal regarding reintegration into society in a sensible way […] » (Senior Physician 3, FG02)
The meso level	
Q5	« I think that we have made great progress at the clinic over the years in discussing the goals with the patients. A few years ago, it was impossible for us to discuss the goals in an interdisciplinary, interprofessional way, where for example, a psychologist openly says in the room, in front of the patient, in front of the whole team, these are the goals. That was not possible a few years ago. » (Occupational Therapist 2, FG03)
Q6	« I feel like these rounds put the patient back in the center. They also have a say. » (Social Worker 2, FG02)
Q7	« I find that they are then much more actively involved. And then some of them think, ok, it's goal setting rounds again today, what is the next goal. » (Psychologist 3, FG02)
Q8	« I think the goal setting round is a very important factor, I think we have much fewer dissatisfied patients […] I personally find that we have much less of that, as I experienced it in the past. I think that the patient sees that we talk to each other, that the doctor also knows about the therapies. […] » (Physiotherapist 2, FG02)
Q9	« […] That's why I find the goal setting meetings with relatives that take place more often with these, how shall I say, “problem patients”, I find that the process is often more pleasant than with “non-problem patients”, where we perhaps forget to have a goal setting meeting with relatives […] » (Physiotherapist 2, FG02)

### Category 3: The micro level

HCP explored and discussed the direct encounter and interaction between HCP and individual patients. In analyzing the FG, three subcategories emerged: knowledge, emotions, and communication. An overview of the themes that were considered most relevant in relation to the subcategories can be found in the Supplementary Table 4.

#### Subcategory 1: Knowledge

HCP highlighted aspects of their own knowledge and that which they share with the patients in order to achieve a successful GSP.

Participants described the importance for each professional group to comprehensively understand the patient's diagnosis. This knowledge allowed HCP to make an initial judgment about the expected outcome, which gave them an idea of possible next steps (Q10). Additionally, HCP emphasized the importance of knowing patients in as much detail as possible. Patients' background, preferences, and general characteristics (e.g. age, educational background, civil status) could further influence the course of rehabilitation (Q11) or reveal helpful prior experiences with GS (Q12). HCP felt that this knowledge helped them when communicating with a patient.

HCP emphasized that patients often lack a general understanding of their situation, especially at the beginning of rehabilitation. In order to guide their patients, HCP needed to have a comprehensive understanding of the functioning that patients may be able to pursue (Q13). However, in addition to guiding patients, HCP should also provide ongoing education to them. Patients' knowledge of their own situation was highlighted as one of the main prerequisites for a successful GSP (Q14). This was not an easy task, especially because patients cannot easily understand their situation and the patient's path is never absolutely predictable. HCP emphasized that they should recognize patients who accept this uncertainty only by gaining knowledge during their initial rehabilitation (Q15) (see Table 2).

**Table 2:** The micro level - knowledge.

Knowledge	
Q10	« I actually do it in such a way that I primarily look at the diagnosis of the patient, what is his diagnosis, what is his ASIA (severity and level of lesion) status, is he a paraplegic, tetraplegic and then I look closely at the expected outcomes. » (Occupational Therapist 2, FG03)
Q11	« […] A 20-year-old patient with multiple comorbidities, the course of her initial rehabilitation will be completely different from a 20-year-old who was an athlete and had an accident due to sports. The athletes also have a slightly different attitude, they are from sport with character, are differently attuned to life and the rehabilitation or the rehabilitation process is then also different […] » (Resident 2, FG01)
Q12	«I think age is very important […] we have a lot of older patients […] I think they just didn't grow up with this culture of setting goals. Now, younger people, during their education, they have to set goals all the time […] » (Nurse 6, FG05)
Q13	« […] I have to have clear goals for myself, because patients very often don't know what goals they can actually achieve. We notice this very often in the ICF goal setting rounds, when we ask what goal you have, the patients shy away, “I don't have a goal, I don't know what I can achieve”. I believe that the staff themselves must have the goals clearly in mind. » (Occupational Therapist 2, FG03)
Q14	« […] the first thing that comes to my mind is that for me, acting in terms of goal setting also has a lot to do with conveying information. Because, especially at the beginning, there is so much jungle and the patient doesn't know why he can't move his arm, for example. I believe that in order to get on the right track, a lot of information is needed at the very beginning so that he can understand. I think that is one of the prerequisites for working together. » (Physiotherapist 1, FG03)
Q15	« Both the fact that paralysis have recovered or are recovering in a way that is not entirely predictable, and that there are other […] limitations, changes this clarity that we can provide. And, for the patients, I think that is somehow “cuddly” at the beginning (laughs). Or like, some of them then push for it “but I want to know now” […] later, they say, “now I understand why you told me that, because we didn't know”. They think it's good that people are so honest, the patients say. » (Senior Physician 3, FG02)

#### Subcategory 2: Emotions

Feelings toward intimate topics could significantly interfere with the GSP. For example, unfamiliarity and shame in dealing with the topic of catheterization or bowel management could hinder GS (Q16). Awareness of these hurdles and sensitive handling on the part of HCP was highlighted as extremely important.

Other frequently mentioned relevant emotional issues included building or maintaining hope and dealing with disappointment. HCP suggested sharing information about the expected outcome with caution to maintain hope and avoid discouragement, especially at the beginning of initial rehabilitation (Q17). Most HCP pointed to the challenge that the patients' personal goals were often viewed unrealistic from a professional perspective. This made it difficult to align patients' goals with professional goals (Q18). Especially because patients were encouraged to set their own goals from the beginning of their rehabilitation process. For less experienced HCP this problem with alignment was described as a main trigger of stress and tension (Q19). HCP had to learn manage this tension for example, by hiding their more skeptical attitude toward an overly optimistic patient. One HCP suggested that observing that this discrepancy usually diminishes toward the end of the patient's initial rehabilitation might provide some relief (Q20). Others recommended that taking a step back and trying to deepen understanding of the patient and his or her situation might be helpful, especially for the less experienced HCP (Q21). In general, patience, acceptance, and empathy were emphasized as helpful prerequisites for the GSP (Q22).

Regarding the GSP, HCP emphasized the need to break larger goals into smaller units. This process allowed for small successes to be highlighted before a patient's “big goal” was achieved. In this way, HCP did not have to argue against a patient's “big goal” and it was easier to reach consensus with patients (Q23). This “break-down” reduced feeling of tension and helped them stay closer to a patient's current possibilities (Q24). Participants mentioned that this process also helped keep patients hopeful and kept them from being overwhelmed by the situation (Q25) (see Table 3).

**Table 3:** The micro level - emotions.

Emotions	
Q16	« For example, catheterization and bowel management are always such topics. There are people for whom it is no problem, and for others, it's a too intimate topic, they cannot talk about it, the timing is very individual. » (Nurse 3, FG03)
Q17	« We have experience, we know how realistic it is, what will come […] The expected outcomes, but we can't, shouldn't, don't want to tell the patients so brutally at the beginning. » (Senior Physician 2, FG03)
Q18	« […]they often want to be able to walk again. It is important not to simply say, “no, you can forget that”. You have to leave hope and still not convey an image that they can undoubtedly walk again, but simply say, “we'll see”. Every step they take is a win, but we don't know where the real stop is, do we? » (Psychologist 5, FG04)
Q19	« I sometimes think that the pressure for the therapists as well as for all disciplines is very high. Because basically, therapists mostly have the desire to achieve the patient's goal. That is always our goal; we want to achieve the patient's goal, if possible. I have seen very old patients who have achieved a lot. But now I also have older patients where I think this goal is much too high. A therapist who is perhaps less experienced might therefore put himself under a lot of pressure. » (Physiotherapist 2, FG02)
Q20	« […] that he not only has confidence in others but that he himself endures the stress of this discrepancy (between the HCP goals and the patient's goals) and finds some comfort. Before leaving (discharge from the clinic), this stress is harmonized in some way. The older ones are then, suddenly, saying, “Oh, I'll stop catheterizing myself, forget it, I've practiced it, now it's enough”. And this moment is enough; now we change the goal because we adapt it to age or culture or something. That's good. But it's a real challenge for the staff, I think. » (Senior Physician 3, FG02)
Q21	« […] it takes a lot of leadership […] I have a lot of young staff in my team, very active team members, who often raise the issue, that the patient should be further ahead. Then I try to look at the whole patient with them, often with mind maps, so that they really see it well, then it actually works very well. » (Physiotherapist 2, FG02)
Q22	« […] he (the patient) then has completely different goals, which we know from experience are probably not realistic. To endure this tension sometimes requires patience and energy and yes, empathy - so that you can still work constructively with the patient or have the feeling that you are working WITH him and not WITHOUT him […] » (Occupational Therapist 4, FG05)
Q23	« I try to set short-term goals where I can take him (the patient) along with me. Like this, you always find a consensus with the patient so that you have a common goal. » (Occupational Therapist 4, FG05)
Q24	« Exactly, it creates a reference to reality (the short-term goals), exactly, and you work on something concrete and that helps you to get out of this powerlessness when you can do something. » (Psychologist 4, FG03)
Q25	« I could imagine that setting small goals also protects (the patients) from being overwhelmed, that you set small goals, step-by-step and therefore, also achieve small successes […] Yes, perhaps not to take away hope in some sense. That you just give small steps. » (Resident 1, FG01)

#### Subcategory 3: Communication

Communication played a crucial role, especially on the topic of expected outcome and prognosis. Participants reported that the way they communicated medical knowledge to patients had changed in recent years. As their expertise increased, they said they had seen unexpected progress in individual patients; some had recovered surprisingly well. This had led to a less direct, more open culture of communication that explicitly included feelings of uncertainty (Q26, Q27). More open communication also included not resisting patients setting goals that HCP considered unrealistic. It was acknowledged that some patients feel the need to set a goal (even if it is considered unrealistic) in order to move forward and focus on the next step. HCP therefore needed to develop self-reflection in relation to the patients progress and adapt compassionately. One HCP suggested asking the patient to set a time limit within which they would both assess progress toward what may be an “unrealistic goal” (Q28). Some HCP indicated that they addressed the problem of unrealistic goals by ensuring that there was a second goal that HCP considered relevant and achievable for the patient. This helped patients experience at least some success and reduced the pressure to achieve the large goals in short time (Q29). More experienced HCP accepted these goals and integrated them into rehabilitation management while internally orienting patients toward a more realistic outcome (Q30). If patients could not yet imagine or set goals for themselves, HCP recommended giving them a choice of realistic goals. This would help patients become comfortable with the rehabilitation process and develop a perspective with them being in the driver's seat (Q31, Q32).

Participants confirmed that the interprofessional environment is relevant to optimizing the GSP. Mutual awareness of goals other professionals had in mind seemed to resonate well with the patients. That was also highlighted to increase efficiency and quality in achieving goals (Q33). Participants felt it was critical to share goals transparently across the IPT, as this helped HCP and patients focus and thus achieve those goals more quickly (Q34). Furthermore, it was highlighted that interprofessional sharing helped HCP gather new ideas on how to address specific goals and how to deal with difficult decisions (Q35).

Lastly, HCP agreed that it is important to become skilled at formulating goals well (Q36). They emphasized that they should be specific and set a realistic timeframe for the goal (Q37) (see Table 4).

**Table 4:** The micro level - communication.

Communication	
Q26	« […] the goals are related to the prognosis. And there is a change in the way one (the HCP) communicates on the medical level, that one rather holds back and says less about what is happening (regarding the prognosis). And that is a cultural decision. It is not primarily the injury pattern, but it is a cultural decision because we have seen that people have always recovered. » (Senior Physician 3, FG02)
Q27	« I think this is more the new culture. A few years ago, people communicated rather stringently that this is this paralysis, and that's how it will be. I think we then noticed that patients often said, “I’ve lost my hope”. That's why this change came about, that you just stay more open. Isn't it? And I think, yes, that certainly helps in recovery. That you don't say so clearly that this is how it is now, but that you leave it open. » (Social Worker 2, FG02)
Q28	« I think it's important to discuss this with the patients, to try it (the patient's goal) out and to set a time limit, so to speak, where we say okay, we will try this for four weeks […] then we will check it and then we will stop it again. Because I think that in this case, sometimes it can help to deal with the disease or with the accident, in order to be able to go one step further than if you had never tried it. » (Physiotherapist 1, FG03)
Q29	« […] that's why I think that these goals are essential, even if they don’t align with the patient's goal at first. But the patient learns while he is here what these goals are. And then he also notices, right, yes; I can achieve that. That is important that he notices that there are things to accomplish. If we let him go with the goal to be able to walk again, and then there is nothing in between, at some point a depression will probably occur […] » (Psychologist 4, FG03)
Q30	« But I think each of us has also experienced the situation that the patient has goals of which we are not convinced or of which we know they are irrational, […] we listen to this patiently at the goal setting rounds […] We work and act in a certain way towards this goal and incorporate it, but we also say to ourselves that we must pursue another goal, which we just think will be important for the future, which may not have been named by the patient […]» (Senior Physician 2, FG03)
Q31	« […] it (the suggested goal) is also a road sign that we can offer to the patients […] when so much is coming up, what is most important at the moment, and what still has a little bit of time, or what we are working towards. » (Physiotherapist 1, FG03)
Q32	« I also find it important to be transparent, sometimes the patient is not yet or not again able to set goals. It is then important to show the patient a perspective of possible goals, give a selection, or show possibilities. » (Psychologist 3, FG02)
Q33	« […] I think it's very important that we (the IPT) always work together. Because there is only one way to achieve a lot of things better, it's if you know exactly who is doing what and the patient also knows, aha, they know from each other how things will continue. » (Social Worker 2, FG04)
Q34	« […] with the concrete goals, since we've been doing it this way, people work much more concretely on the goals and often work more consciously on the goals […] and achieve them quicker since we've really made it so transparent. And I also find the appearance as an interdisciplinary team at the rounds, with the precise wording of goals. I also find that what I experience, I'll put it this way now, impresses the patients. They are much more aware of the goals they are working towards. » (Nurse 3, FG03)
Q35	« For me personally, it's always a highlight during my work when something doesn't work, and then you work together or exchange ideas with another professional group and when he (the patient) can do that, and you can see that he enjoys it, that there is a solution for him, that brings me a lot of joy. » (Physiotherapist 1, FG03)
Q36	« I would also tell them (the HCP) to learn to word goals well. I think this is highly underestimated, it is also a demanding task to phrase goals. » (Psychologist 1, FG05)
Q37	« […] we should actually word the goals a little more precisely when we say catheterize, that is to say, which step of catheterization do we want to look at in the next two weeks? And just, in a fortnight, what can you realistically do by then? And do we also check it? I think it is important to define the goal much more precisely. » (Occupational Therapist 4, FG05)

## Discussion

The results showed that HCP identified GSP-relevant aspects at three different levels: at the level of national systems and regulations, at the level of organizational culture and structural elements of the clinic, and at the individual level in relation to the direct interaction between HCP and patient. HCP mentioned the need to acquire specific knowledge and skills at the micro level. This included expertise and experience to assess the patient's situation and the expected outcome of the GSP. In addition, they needed to be able to establish the emotional foundation necessary to interact well with the patient and other HCP during GS. Acceptance of the patients' path and their goals developed as an emotional skill and a prerequisite for good interaction with patients while managing stress and tension. Finally, HCP emphasized solid interprofessional collaboration and communication skills to educate patients and accept their journey. Mastery of all these points can reinforce a truly patient-centered perspective and favor shared decision making. Ultimately, this can lead to more frequent adherence to goals, better rehabilitation planning, and increased patient and HCP satisfaction.

Participants identified influential macro-level aspects, such as legal or policy features of the healthcare system that affect rehabilitation by influencing service delivery or financing (30). It is important to report standardized outcome measures based on for example, the ISCOS toolkit (31) or the Nottwil Standard (23). These outcome measures form the basis for documenting rehabilitation outcomes in terms of rehabilitation duration and can then be used to make informed decisions about appropriate service delivery. In addition, they are needed to calculate rehabilitation trajectories that can provide information about expected outcomes and lead to better rehabilitation planning (32).

At the meso level, established collaborative structures such as IPT meetings were highlighted as extremely useful for the GSP. This should remain an integral part of any rehabilitation, consistent with previous literature (33). Structures and standardized processes therefore contribute to the quality of rehabilitation, and are also a requirement in certification programs such as the Swiss Reha quality criteria (34). In addition, regular meetings allow goals to be tracked, adjusted as needed, motivate by showing progress, and ensure that meaningful goals are the focus of rehabilitation. Together with formulating action plans, identifying inhibiting and facilitating factors, and assessing patient's confidence in achieving goals, these are the relevant GS intervention components identified in the literature (35).

At the micro level, patients set goals in consultation with HCP. To do this, they must be able to correctly assess their health status. Therefore, it is important to empower patients and promote their health literacy through education (13). The type of education needs to be adjusted over time: at the beginning, more support is needed with clear examples of possible goals. Toward the end, patients become experts on themselves and less guidance and more of a partnership is needed, where HCP provide medical expertise (36). The timing of this change varies. Some patients need more time than others to understand and accept their new situation. Consequently, an important skill for GS is to accept the patient's journey and pace so that they can slowly reframe their goals into more realistic ones during the course of rehabilitation (37).

However, it was a great challenge for HCP to endure the tension between their own rehabilitation plan with corresponding goals and unrealistic patient expectations, which led to stress (38). Patients' goals often reflected their long-term hopes, while HCP preferred to focus on a more realistic short-term goal (39, 40). Because patient outcomes cannot be predicted with absolute certainty (41) patients and HCP must manage the uncertainty of the process. Therefore, HCP need to accept patients' hope as part of reality and improve their skills in listening, negotiating and building partnerships (42). This shows that communication is not just about conveying information but must also include aspects of patient-centered communication. These aspects include being sensitive to and accepting of the patient's needs, lived experiences and desires in relation to their goals (43). While negotiating goals was the first part of GS in the older literature (19), our participants suggested listening to and accepting the patient's goals. HCP focus on the patient's self-efficacy by converting goals into smaller, achievable goals without negotiation until consensus is reached.

Participants talked about another important GS aspect at the individual level: goal formulation. Although the SMART framework is well known in the rehabilitation center and HCP explicitly stated that goals should be realistic within a certain time-frame, there are important goals that cannot always be formulated in this way (e.g. psychological growth). Therefore, while these criteria are supportive they are not sufficient to achieve a better GSP (16). In addition, the application of SMART criteria in SCI/D rehabilitation is challenging because this framework requires clarity and specificity regarding the end result to be achieved. As mentioned earlier, progress in SCI/D tends to be uncertain, and therefore, goals are process-oriented rather than SMART as defined (44).

### Strengths and limitations

One strength of the study was the quality of the thematic inductive process, which included independent thematic analyses by two researcher and several critical discussion sessions with all experts involved.

Another strength, but also constraining aspect, was the communication training HCP had received for years. Based on this cultural development, HCP may had been able to recognize this spectrum of influencing aspects, including patients' development and personal growth. Considering that this was a single-center study, the results should be viewed as an example of a clinic that places a high value on communication and patient-centeredness. However, to reduce sample bias and enhance gathering of different perspectives, it would have been beneficial to include other SCI/D rehabilitation centers.

In addition, the participant sample included small subsamples of different professions that were somewhat unevenly represented. This means that some professions were able to provide more information for analysis than others. Furthermore, the majority of participants were in leading positions. This may also have affected the results, as the perspectives of less experienced HCP were less represented. The advantage of this participant selection was the broad and experienced background, which led to a deeper understanding of rehabilitation. Consequently, these results may provide the basis for future improvements or specific trainings for the GSP in initial rehabilitation, particularly assisting less experienced HCP.

### Recommendation for further development

Aspects at the macro, meso and micro levels should be considered when thinking about how to improve GS during initial rehabilitation after newly acquired SCI/D. The duration of rehabilitation is crucial to give the patient and the IPT sufficient time to adapt to the new living situation. Time for IPT exchange is necessary to allow effective IPT collaboration. At the micro level, specific training especially for inexperienced HCP should include: dealing with the uncertainty of expected outcomes, the different characteristics of goals (specific and measurable or nonspecific and timeless), the patients' personal adjustment process, and the challenges of communication and emotional encounters with the patient. To our knowledge, these aspects are rather unexplored in GS research, so further investigation is needed. In addition, audit research examining goal achievement in the clinic over a 12- to 18-month period could be useful to better understand topic-related challenges to the GSP. After development and implementation of a specific training program, a follow-up study of patient satisfaction could be planned to show the potential effectiveness of the intervention. Nonetheless, the results of this study may help HCP develop innovative strategies for complex GS situations. They make a main contribution to GS practice in settings with complex chronic conditions, which is of utmost importance to assure the expected beneficial outcomes during rehabilitation.

## Data Availability

The raw data supporting the conclusions of this article will be made available by the authors, without undue reservation.
