# Supplementary material for: Health care professionals’ experiences with goal setting during initial rehabilitation after newly acquired spinal cord injury/ disorder – a qualitative focus group study
Source: Front Rehabil Sci. 2022 Aug 18;3:982321. doi: 10.3389/fresc.2022.982321 (PMC9397958; doi:10.3389/fresc.2022.982321)
Supplement: Supplementary file 1 [file Table_1_v1.docx]

Supplementary Material

**Table 1. FG guide**

| **Introduction** | - Recording starts now  1. We start with the person on my left and ask you to continue clockwise. Please name your: *Name, profession, years in profession, years in the clinic* |
| --- | --- |
| **In general** | Goal setting is an essential component in rehabilitation.   1. What comes to your mind spontaneously when you think of goal setting in initial rehabilitation after newly acquired SCI?    - *What influences goal setting?*    - *How do you set goals?*    - *According to which criteria, with which considerations, etc.?* |
| **Patient-specific factors** | 1. We have already heard some topics that concern patient-specific factors. So, we would first go into that. Patient specific factors – could you tell us more about those?    - *Family, financial situation, level of education?*    - *Different types/preferences?* |
| **Factors from HCP's perspective** | 1. It has already been mentioned that you, as health care professionals, have an influence on the framing of the goal setting process through your actions. Can you tell me something about this?  - *Responding to patient's needs? Promote participation?* - *Facilitators/ barriers?* - Changed behavior over the course of initial rehabilitation? |
| **General conditions** | 1. During the discussion you could already hear that certain framework conditions at work have an influence. Could you tell me more about this?    - *Ideal framework conditions for the goal setting process 🡪 How?*  - *Aids/tools as recommendation or wish?* |
| **Closing** | 1. Thank you very much for all your contributions. Finally, we would like to ask you what specific ideas you have for improving the goal setting process? |
| - Unless anyone else wishes to comment, the recording will now be stopped.   Thank you for your input and contributions. | |

**Table 2. Overview FGs**

| FG 1 | Resident 1, Resident 2, Nurse 2, Psychologist 2 |
| --- | --- |
| FG 2 | Senior Physician 3, Physiotherapist 2, Psychologist 3, Social Worker 2 |
| FG 3 | Senior Physician 4, Senior Physician 2, Nurse 3, Occupational Therapist 2, Physiotherapist 1, Psychologist 4 |
| FG 4 | Occupational Therapist 3, Physiotherapist 3, Psychologist 5, Social Worker 2 |
| FG 5 | Senior Physician 1, Nurse 5, Nurse 6, Occupational Therapist 4, Psychologist 1 |

**Table 3. Participant characteristics**

| **Characteristics** | **Sample N=22** |
| --- | --- |
| Sex | |
| Male | 4 (18%) |
| Female | 18 (82%) |
| Profession | |
| Nurse | 4 (18%) |
| Occupational therapist | 2 (9%) |
| Physician | 6 (27%) |
| Resident | 2 (9%) |
| Senior Physician | 4 (18%) |
| Physiotherapist | 4 (18%) |
| Psychologist | 5 (23%) |
| Social worker | 1 (5%) |
| Years in the clinic | |
| <5 years | 6 (27%) |
| 5-10 years | 11 (50%) |
| 11-20 years | 3 (14%) |
| >20 years | 2 (9%) |

**Table 4. Overview subcategories micro level**

| **Subcategory** | **Theme** |
| --- | --- |
| Knowledge | Knowing the expected outcome and therefore possible goals |
|  | Knowing and considering patient-specific factors |
|  | Educating the patient regarding the expected outcome |
| Emotions | Preserving hope |
|  | Withstanding the tension of GS |
| Communication | Communication culture |
|  | Collaborating with the patient during GS |
|  | Approaching GS with interprofessional collaboration |
|  | Achieving the best possible wording of the goals |
